# Author Correction: Maximizing carbon sequestration potential in Chinese forests through optimal management

**DOI:** 10.1038/s41467-024-48054-1

**Published:** 2024-05-13

**Authors:** Zhen Yu, Shirong Liu, Haikui Li, Jingjing Liang, Weiguo Liu, Shilong Piao, Hanqin Tian, Guoyi Zhou, Chaoqun Lu, Weibin You, Pengsen Sun, Yanli Dong, Stephen Sitch, Evgenios Agathokleous

**Affiliations:** 1 Key Laboratory of Ecosystem Carbon Source and Sink, China Meteorological Administration (ECSS-CMA), School of Ecology and Applied Meteorology, Nanjing University of Information Science and Technology, Nanjing, 210044 China; 2 Key Laboratory of Forest Ecology and Environment, China’s National Forestry and Grassland Administration, Ecology and Nature Conservation Institute, Chinese Academy of Forestry, 100091 Beijing, China; 3 Key Laboratory of Forest Management and Growth Modelling, China’s National Forestry and Grassland Administration, Research Institute of Forest Resource Information Techniques, Chinese Academy of Forestry, 100091 Beijing, China; 4 Forest Advanced Computing and Artificial Intelligence Laboratory (FACAI), Department of Forestry and Natural Resources, Purdue University, West Lafayette, IN 47907 USA; 5 College of Forestry, Northwest agriculture and Forestry University, Yangling, 712100 China; 6 Sino-French Institute for Earth System Science, College of Urban and Environmental Sciences, Peking University, 100871 Beijing, China; 7 Schiller Institute for Integrated Science and Society, Department of Earth and Environmental Sciences, Boston College, Chestnut Hill, Massachusetts, MA 02467 USA; 8 Department of Ecology, Evolution, and Organismal Biology, Iowa State University, Ames, IA 50011 USA; 9 College of Forestry, Fujian Agriculture and Forestry University, Fuzhou, 350002 China; 10 College of Life and Environmental Sciences, University of Exeter, Exeter, UK

**Keywords:** Forestry, Carbon cycle

Correction to: *Nature Communications* 10.1038/s41467-024-47143-5, published online 11 April 2024

The original version of this Article contained an error in Fig. 1, which did not represent fully updated data. This has been corrected in both the PDF and HTML versions of the Article.

The original version of the Supplementary Information associated with this Article had identical information presented in Tables S2 and S3, which has been corrected to remove the redundant information. The HTML has been updated to include a corrected version of the Supplementary Information.

### Supplementary information

Updated Supplementary Information

