# Supplementary material for: Maximizing carbon sequestration potential in Chinese forests through optimal management
Source: Nat Commun. 2024 Apr 11;15:3154. doi: 10.1038/s41467-024-47143-5 (PMC11009231; doi:10.1038/s41467-024-47143-5)
Supplement: Supplementary file 1 — Supplementary Information [file 41467_2024_47143_MOESM1_ESM.pdf]

Supporting Information for

Maximizing carbon sequestration potential in Chinese forests through optimal management

Zhen Yu[1,2]*, Shirong Liu[2]*, Haikui Li[3], Jingjing Liang[4], Weiguo Liu[5], Shilong Piao[6], Hanqin Tian[7], Guoyi Zhou[1], Chaoqun Lu[8], Weibin You[9], Pengsen Sun[2], Yanli Dong[1], Stephen Sitch[10], Evgenios Agathokleous[1]

Affiliations:

[1]Key Laboratory of Ecosystem Carbon Source and Sink, China Meteorological Administration (ECSS-CMA), School of Ecology and Applied Meteorology, Nanjing University of Information Science and Technology, Nanjing, 210044, China.

[2]Key Laboratory of Forest Ecology and Environment, China's National Forestry and Grassland Administration, Ecology and Nature Conservation Institute, Chinese Academy of Forestry, Beijing, 100091, China.

[3]Key Laboratory of Forest Management and Growth Modelling, China's National Forestry and Grassland Administration, Research Institute of Forest Resource Information Techniques, Chinese Academy of Forestry, Beijing, 100091, China

[4]Forest Advanced Computing and Artificial Intelligence Laboratory (FACAI), Department of Forestry and Natural Resources, Purdue University, West Lafayette, IN, 47907, USA.

[5]College of Forestry, Northwest agriculture and Forestry University, Yangling, 712100, China.

[6]Sino-French Institute for Earth System Science, College of Urban and Environmental Sciences, Peking University, Beijing, 100871, China.

[7]Schiller Institute for Integrated Science and Society, Department of Earth and Environmental Sciences, Boston College, Chestnut Hill, MA, 02467, USA.

[8]Department of Ecology, Evolution, and Organismal Biology, Iowa State University, Ames, IA, 50011, USA.

[9]College of Forestry, Fujian Agriculture and Forestry University, Fuzhou, 350002, China.

[10]College of Life and Environmental Sciences, University of Exeter, Exeter, UK

*Corresponding author. Email: zyu@nuist.edu.cn; liusr@caf.ac.cn

This PDF file includes:

Supporting text
Figures S1 to S18
Tables S1 to S9
SI References

Supporting Information Text

Spatial distribution of the forest plots surveyed during national forest inventory

About 415, 000 permanent plots were set up in China for routine survey each five years. Among these plots, nearly 30,000 to 50,000 surveyed plots were covered with trees during the period of the 6th (1999-2003) to 9th NFI (2014-2018) (Figure S1). For each forest plot, trees with diameter at breast height (DBH) ≥ 5cm were labeled, measured, and recorded. In total of 18,116,071 tree were recorded, and the tree volume was calculated by referring to the one-variable tree volume tables for each species specifically developed in each province (Ministry of Agriculture and Forestry of China, 1978). The stand volumes were summarized to plot level from all recorded trees.

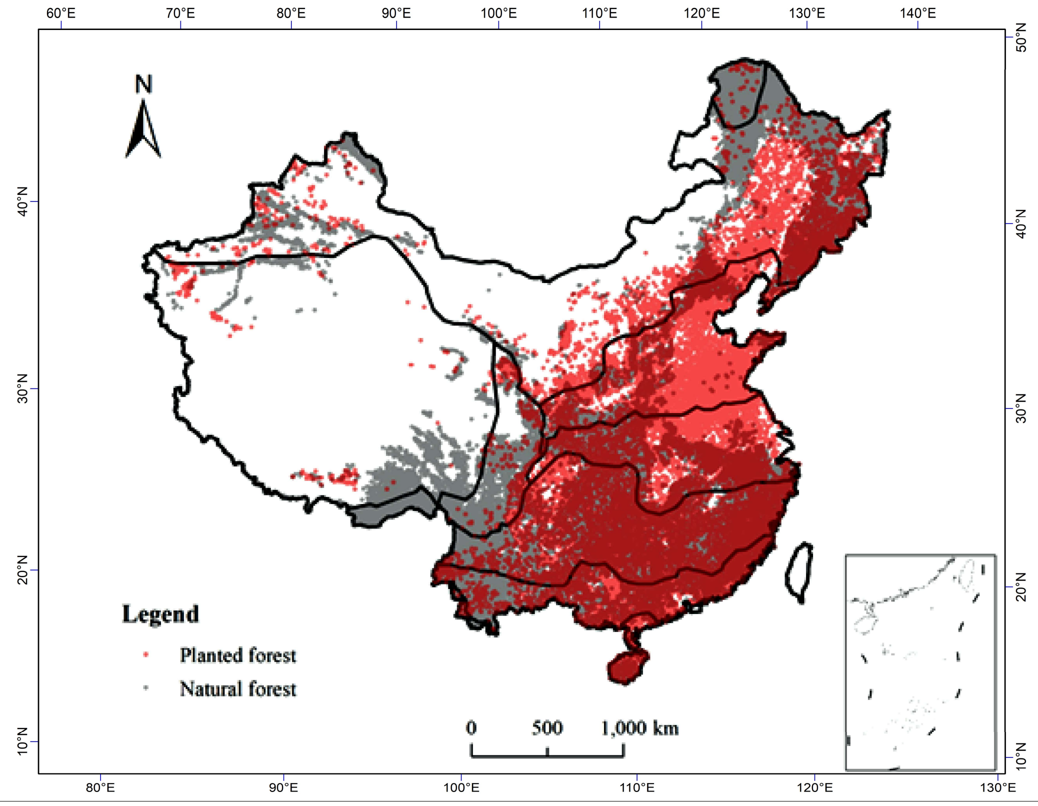

Fig. S1. Locations of the national forest inventory plots in China. Red and gray dots indicate plots of planted and natural forests.

Forest carbon stock and age

We converted the tree volume data into biomass carbon stock using the continuous biomass expansion factor method (Table S1) and the species-specific carbon conversion parameters (Tables S2). We examined the biomass carbon stock changes with stand age in natural and planted forests (Figure S2). As expected, forest carbon stocks increase with stand age but saturate as they proceed to mature (Figures S2A-C). The relationships of forest biomass carbon and age were used to estimate the species-specific carbon stock in 2018, which serve as the reference for calibration and validation of the simulations using process-based model (DLEM). Besides, the age and biomass carbon for each major tree species were showed in Figure S3.

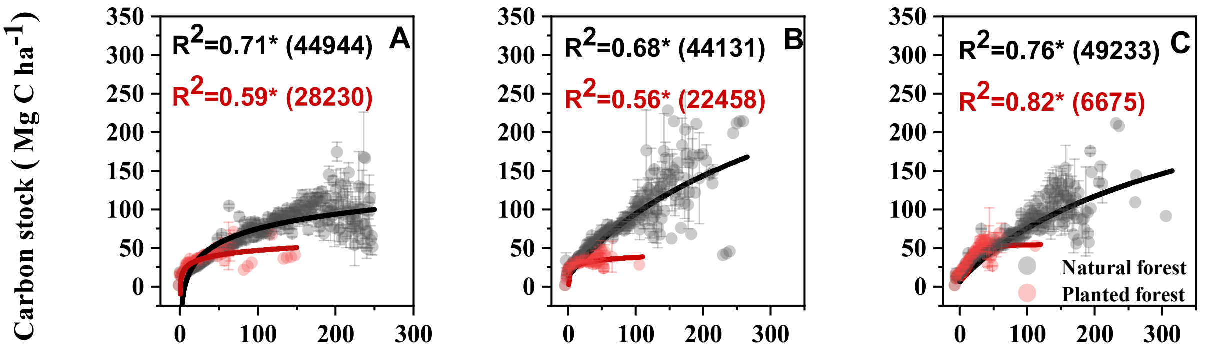

Fig. S2. Relationships between forest biomass carbon accumulations and ages. Panels A-C: relationships of forest stock volume and stand age; error bars indicate 1 standard error from the mean; * indicate significant at p<0.05 level.

The age and biomass carbon relationship of different tree species in needle-leaf forests

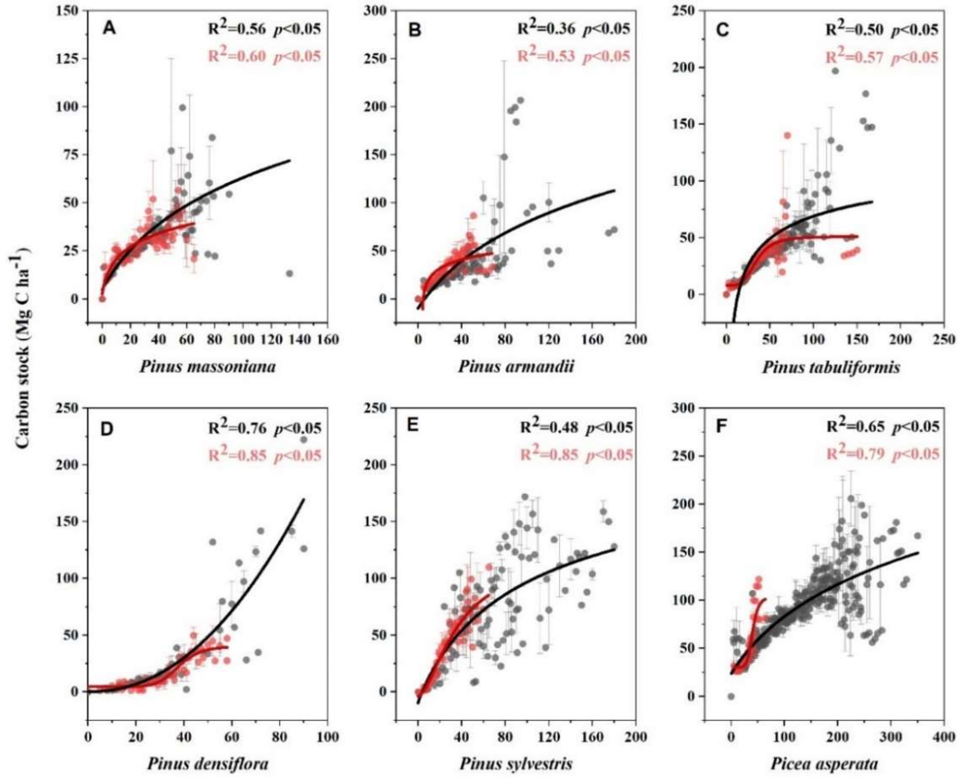

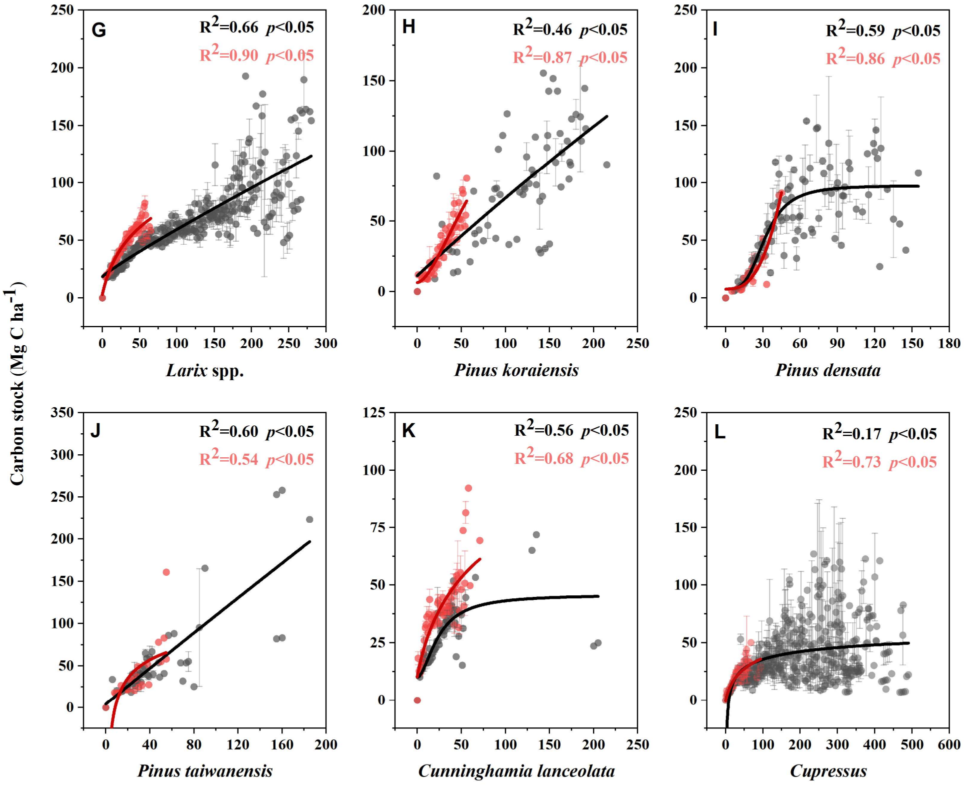

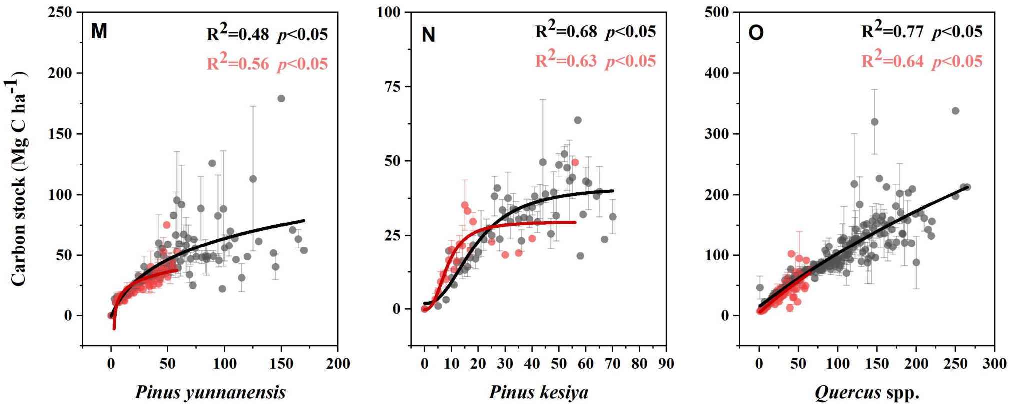

Fig. S3. The age and biomass carbon relationship of different needle-leaf tree species derived from the 6th to 9th national forest inventory data. Panels A-O indicate *Pinus massoniana*, *Pinus armandii*, *Pinus tabuliformis*, *Pinus densiflora*, *Pinus sylvestris*, *Picea asperata*, *Larix spp.*, *Pinus koraiensis*, *Pinus densata*, *Pinus taiwanensis*, *Cunninghamia lanceolata*, *Cupressis*, *Pinus yunnanensis*, *Pinus kesiya*, and *Quercus spp.*, respectively. Error bars indicate the standard error of means.

The age and biomass carbon relationship of different tree species in broadleaf forests

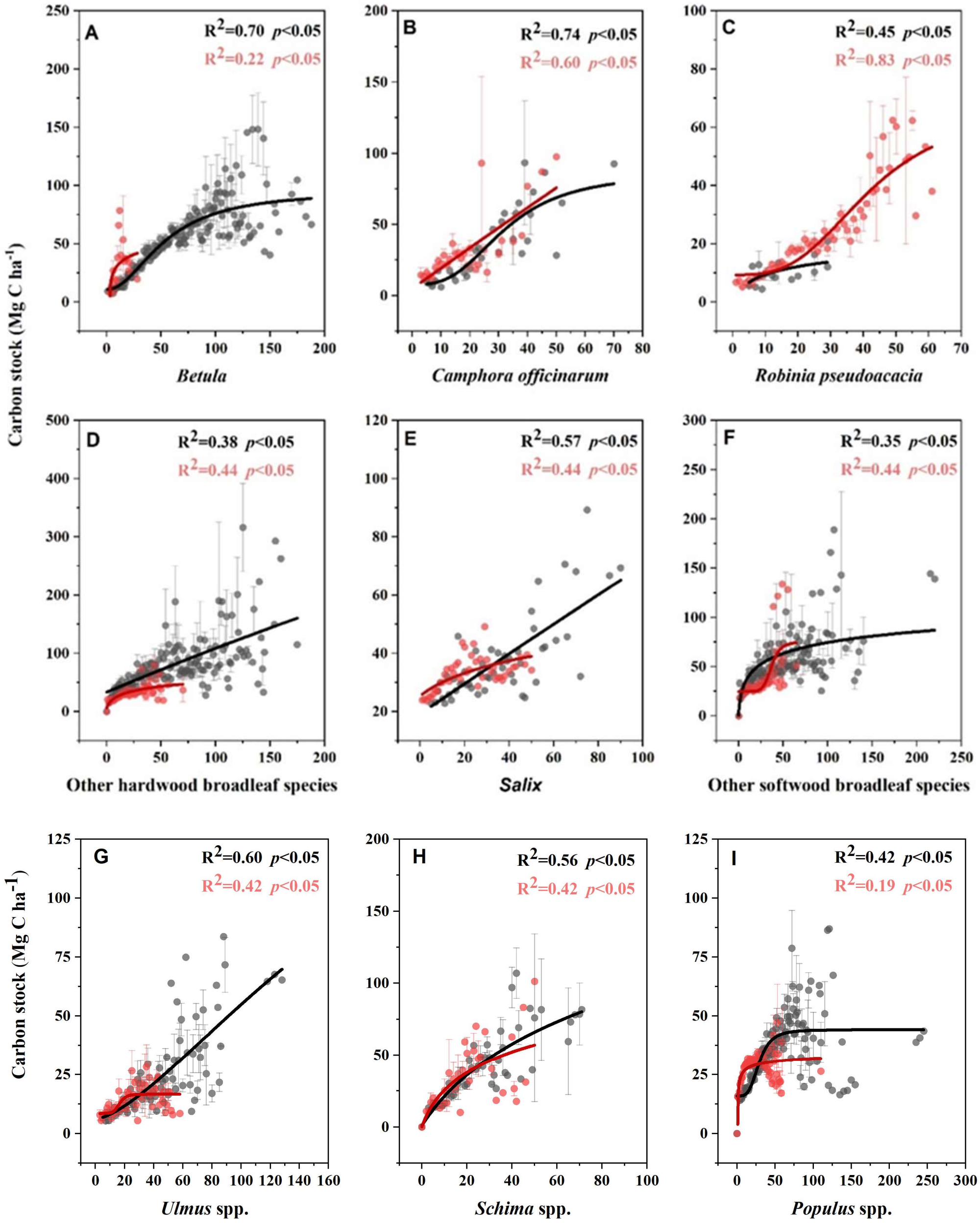

Fig. S4. The age and biomass carbon relationship of different broadleaf tree species derived from the 6th to 9th national forest inventory data. Panels A-I indicate *Betula*, *Camphora officinarum*, *Robinia Pseudoacacia*, Other hardwood broadleaf species, *Salix*, Other softwood broadleaf species, *Ulmus spp., Schima spp.,* and *Populus spp.,* respectively. Error bars indicate the standard error of means.

The age and biomass carbon relationship in mixed forests

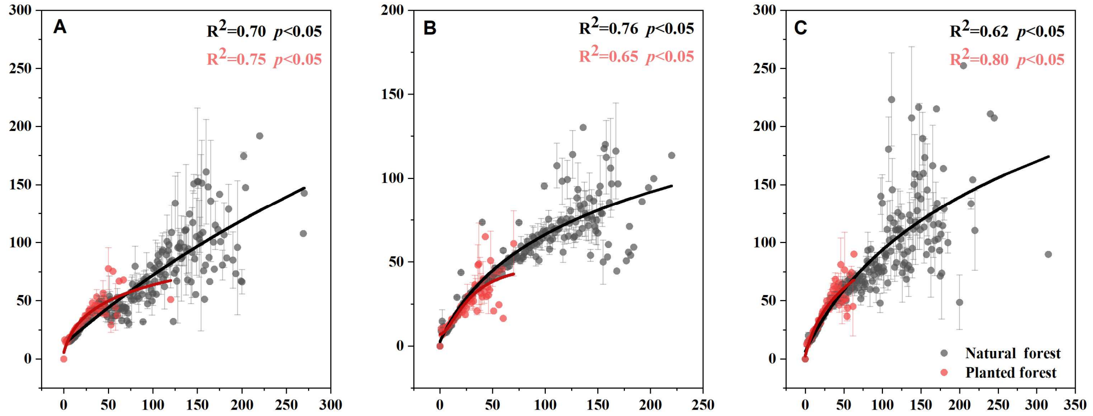

Fig. S5. The age and biomass carbon relationship of different mixed forests derived from the 6th to 9th national forest inventory data. Panel A-C indicate need-leaf mixed forests, broadleaf mixed forests, and need-lead and broadleaf mixed forest, respectively. Error bars indicate the standard error of means.

Forest carbon stock and sink calibration and validation at species level

We compared the forest carbon stock derived from inventory data and process-based biogeochemical model (DLEM) for each species of natural forest and planted forest (Figure S6). We further compared the forest carbon sink in 2020 derived from inventory data and process-based biogeochemical model for each tree species (Figure S14). Specifically, the sink of the no harvesting scenario from the process-based model was compared with the results derived from the statistical model. We also compared the temporal changes in forest biomass carbon sink derived from statistical model and the process-based biogeochemical model under no harvesting scenario (Figure S7).

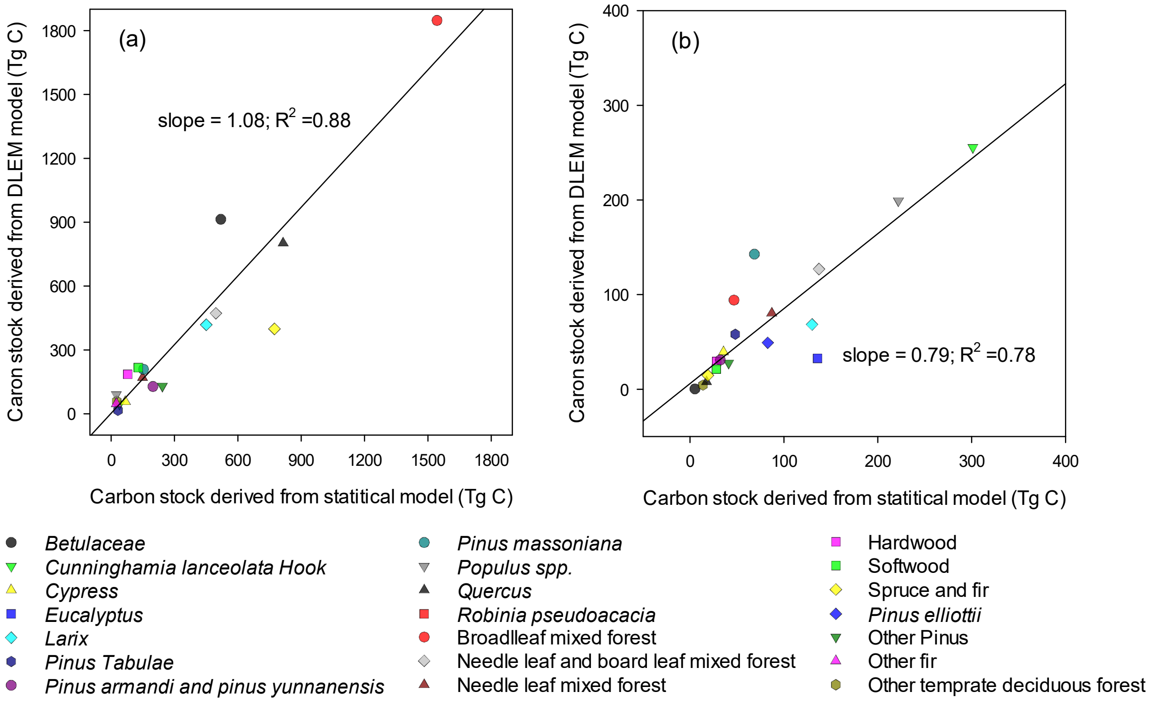

Fig. S6. Comparison of carbon stock derived from statistical model and process-based model at species level in 2018. Panel a: natural forest; Panel b: planted forest.

Comparison of carbon sink derived from statistical and process-based model with exclusion of wood harvest

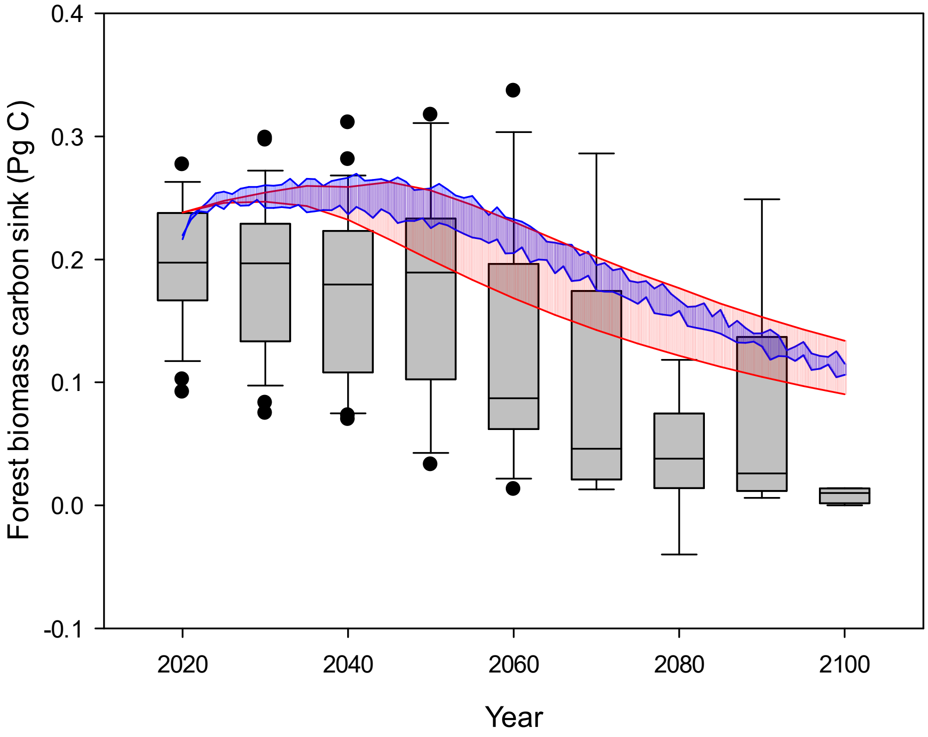

Fig. S7. Comparison of carbon sinks derived from projections with exclusion of wood harvest. Red shaded area indicates the carbon sink derived from statistical model; blue shaded area indicates the carbon sink derived from process-based model (DLEM) with exclusion of wood harvest, climate change, and rising CO_2; boxes indicate the carbon sinks reported in other studies. Results of DLEM simulations were derived from group 3 experiments.

Peak year of the forest biomass carbon sink under different SSPs

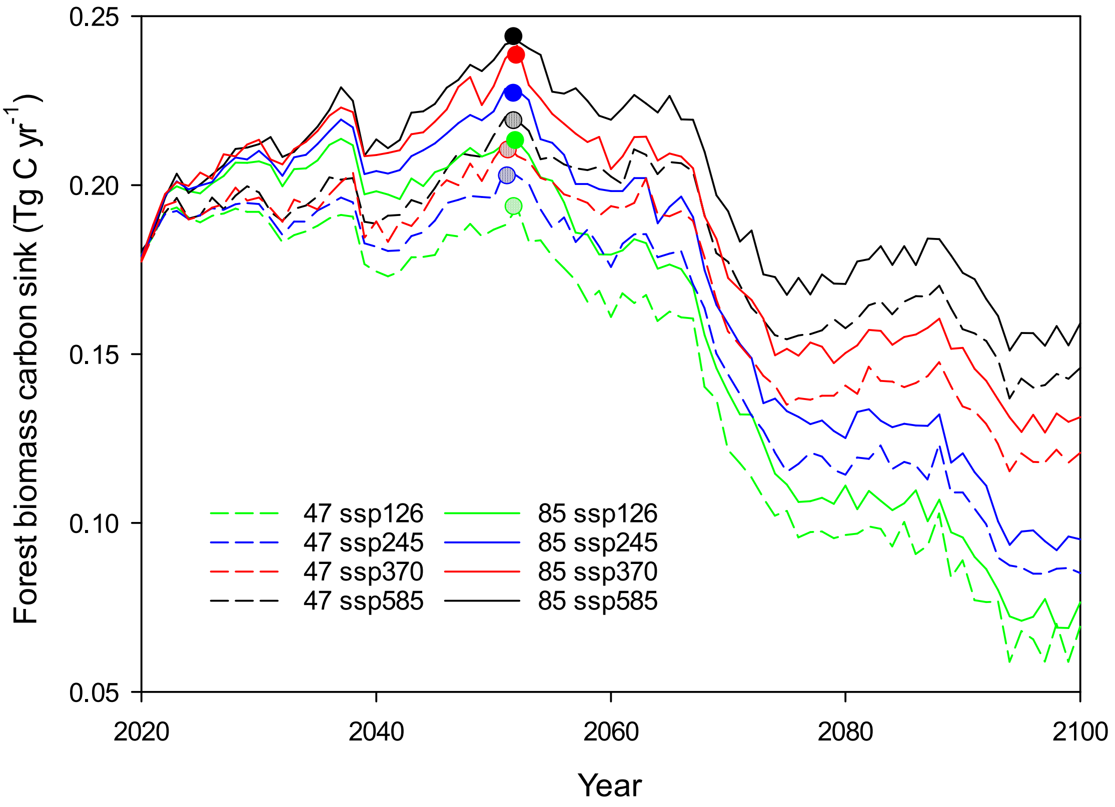

Fig. S8. The modeled biomass carbon sink and the year of sink peak under different scenarios. Dash line and solid line indicate the carbon sink under the scenarios of current tree survival rate (47%) and elevated tree survival rate (85%); ssp126, ssp245, ssp370, and ssp585 indicate simulation of climate and CO_2 under the scenarios of SSP1-2.6, SSP2-4.5, SSP3-7.0, and SSP5-8.5; Dotted circle and closed circle indicate the biomass carbon sink peak under the scenarios of current tree survival rate (47%) and elevated tree survival rate (85%), respectively. Results were derived from group 1 experiments.

Diagram showing forest age change and age impacts on carbon accumulation

Stand age is the average age of trees at canopy level, but average age was derived from all trees in the plot (Figure S9). Generally, stand age was more widely used in statistical model, while process-based models rely on average age. However, process-based model using average age may cause bias in representing the growth rate (Figure 4). For example, Cohort1 and Cohort2 in Figure 4 indicate a young age plot and an aged plot, in which the carbon accumulation rate F_{ave} (instantaneous growth rate, the first-order derivative of the growth curve) at the average age is high, but in reality, the average growth rate (average of F1 and F2) is very low. Thus, age dynamics should be particularly tracked in modeling of tree growth.

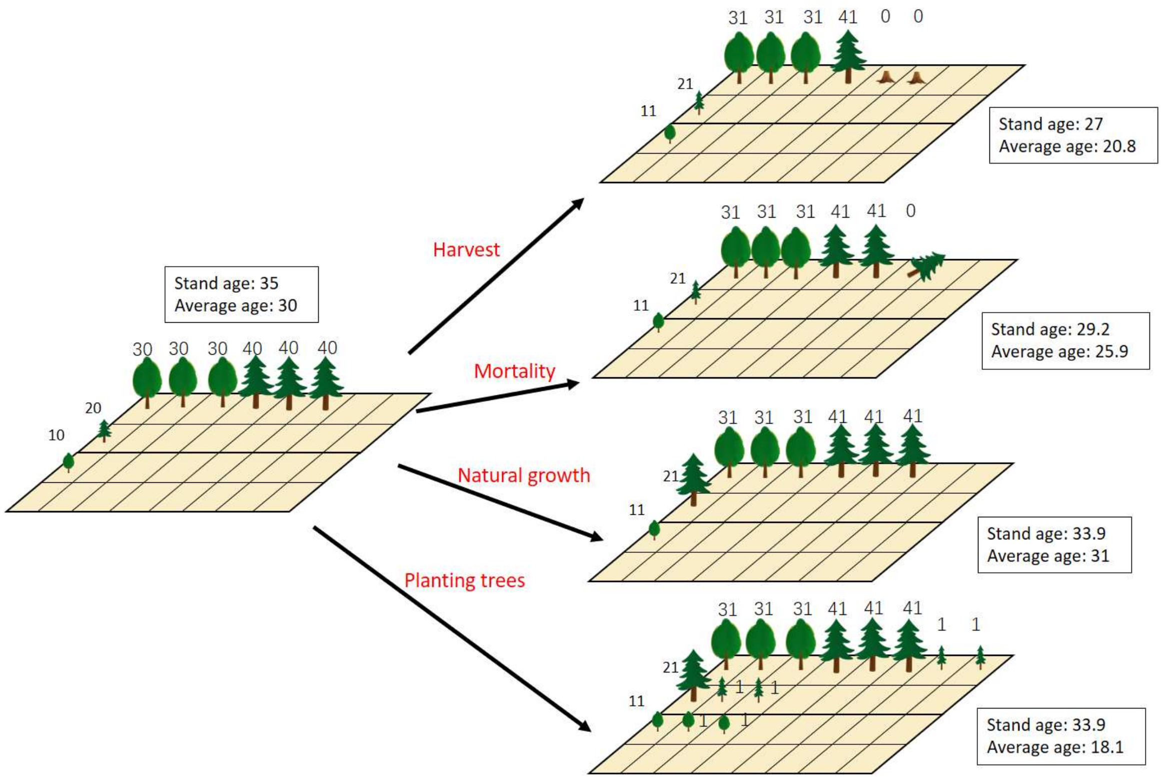

Fig. S9. The dynamic of the stand age and average age in a plot due to wood harvest, mortality, natural growth, and tree-planting. Stand age: average age of the trees in the overstory level; average age: averaged age of all trees in the plot. The numbers above trees indicate the tree ages in the plot.

Peak year of the forest biomass carbon sink under different SSPs assuming no harvest and annual age accrual at 1 year per year

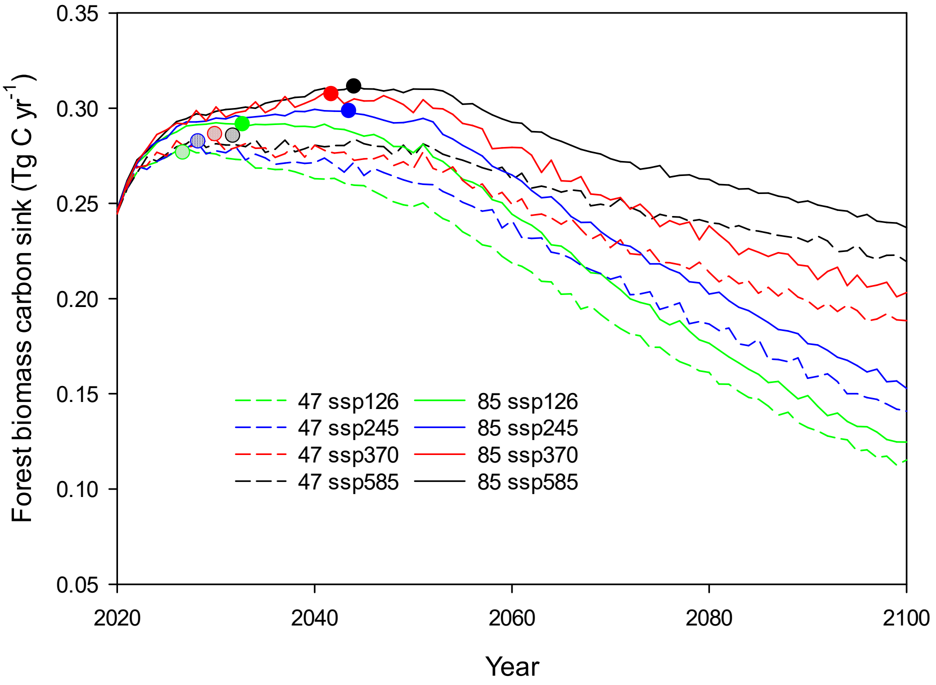

Fig. S10. The modeled biomass carbon sink and the year of sink peak under scenarios assuming no harvest and annual forest age accrual at 1 year per year. Dash line and solid line indicate the carbon sink under the scenarios of current tree survival rate (47%) and elevated tree survival rate (85%); ssp126, ssp245, ssp370, and ssp585 indicate simulation of climate and CO_2 under the scenarios of SSP1-2.6, SSP2-4.5, SSP3-7.0, and SSP5-8.5; Dotted circle and closed circle indicate the biomass carbon sink peak under the scenarios of current tree survival rate (47%) and elevated tree survival rate (85%), respectively. Results were derived from group 3 experiments.

Model simulated biomass carbon removed by wood harvest

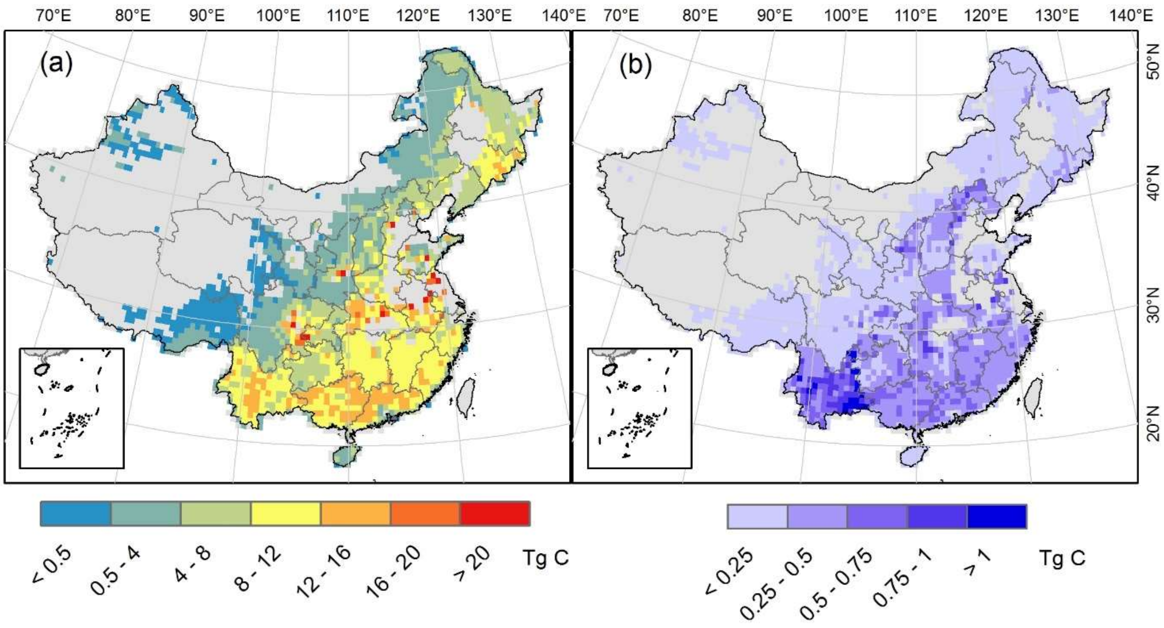

Fig. S11. The modeled biomass carbon removed by wood harvest in China during the period 2020-2100. Panel a: average of wood harvest; panel b: standard deviation of the harvest wood; Unit: Tg C per gird cell. Results were derived from group 1 experiments.

Model simulated forest biomass carbon stock in 2020, 2060, and 2100

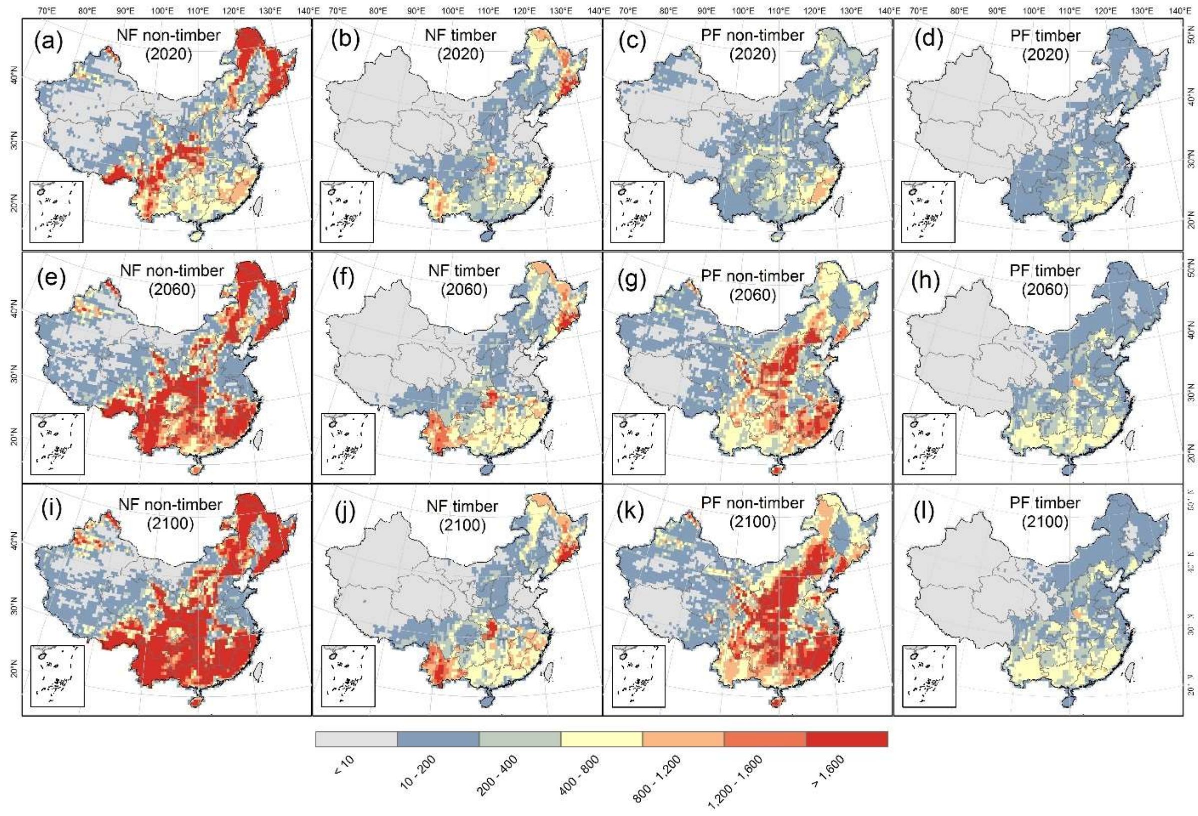

Fig. S12. The projected biomass carbon stock in China's forests. NF non-timber, NF timber, PF non-timber, and PF timber indicate non-timber natural forest, timber natural forest, non-timber planted forest, and timber planted forest; panels a-d, e-h, and i-l indicate the spatial distribution in 2020, 2060, and 2100, respectively; unit: g C m^{-2}. Results were derived from group 1 experiments.

Spatial distribution of the changes in model simulated forest biomass carbon stock during the periods of 2020-2060 and 2060-2100

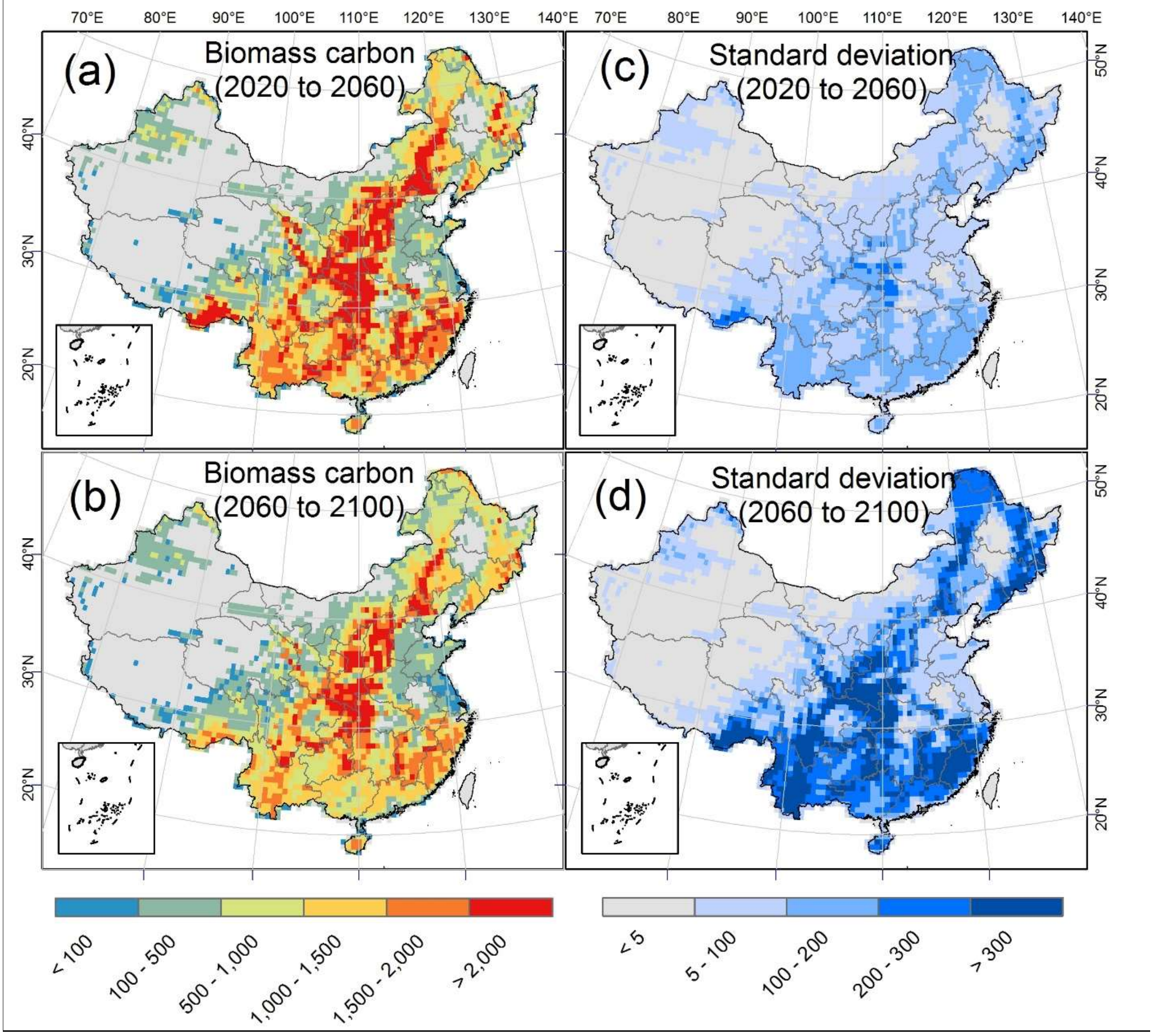

Fig. S13. The modeled biomass carbon stock changes in China. Panels a-b, and c-d indicate the biomass carbon stock change and the standard deviation from 2020 to 2060 and from 2060 to 2100, respectively; unit: g C m^{-2}. Results were derived from group 1 experiments.

Species-specific carbon sink derived from statistical model and process-based model

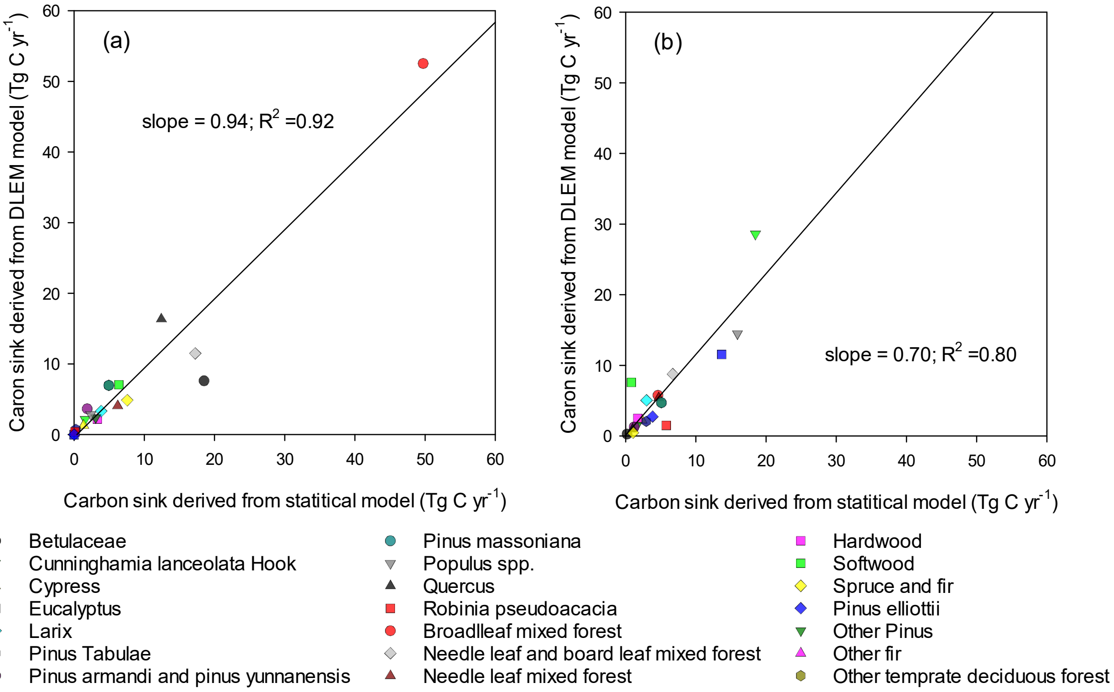

Fig. S14. Comparison of carbon sink derived from statistical model and process-based model at species level in 2020. Panel a: natural forest; Panel b: planted forest.

Spatial distribution of the existing and new forests in China.

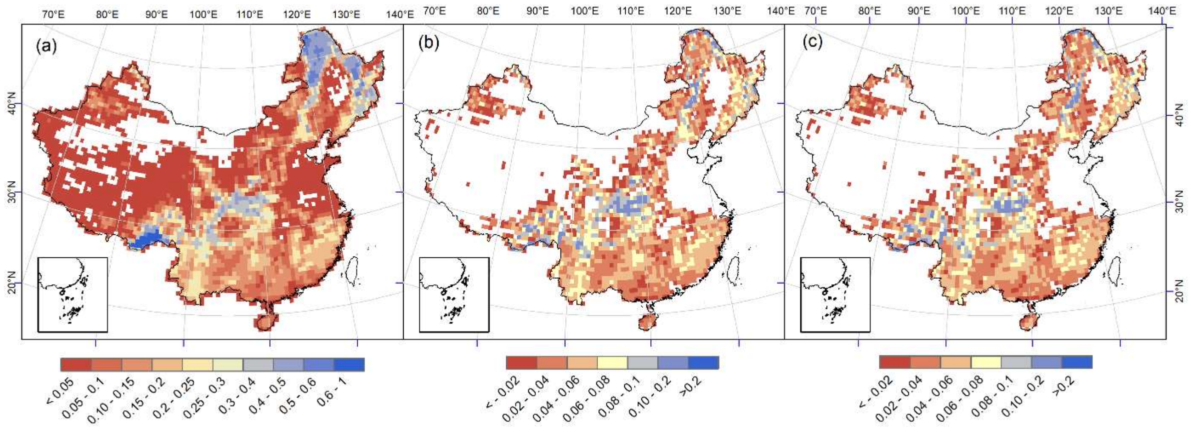

Fig. S15. The distributions of the existing and new forests. Panel a: forest coverage in 2018; panel b: new forests from 2018 to 2100 under a 47% tree survival rate; panels c: new forests from 2018 to 2100 under 85% tree survival rate. The number indicates the coverage percentage of the forests.

Spatial distribution of the habitats suitability index of *Pinus massoniana, Pinus sylvestris,* and *Picea spp.* under climate change.

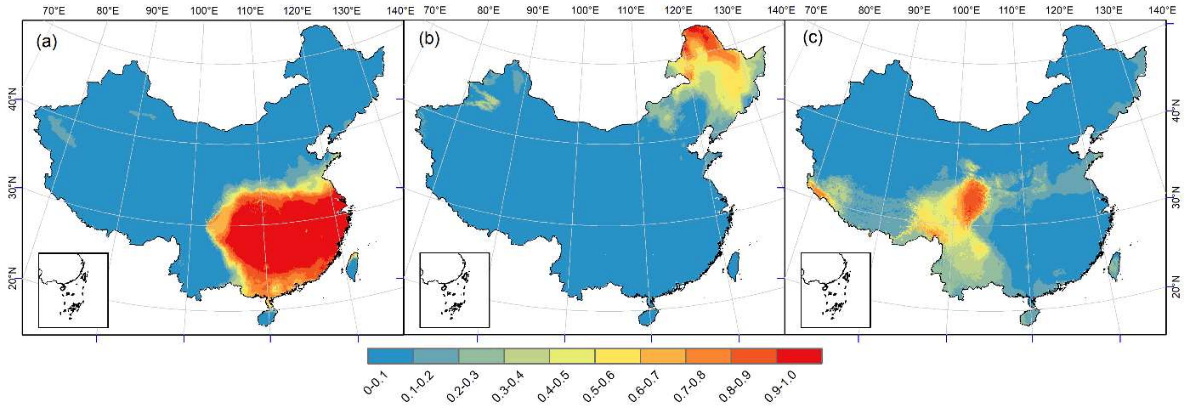

Fig. S16. The suitability index distribution maps of (a) *Pinus massoniana*, (b) *Pinus sylvestris*, and (c) *Picea* spp. in China under future climate change. Suitability index ranges from 0 to 1. Higher value indicates higher suitability for the species.

Spatial distribution of forest management zones

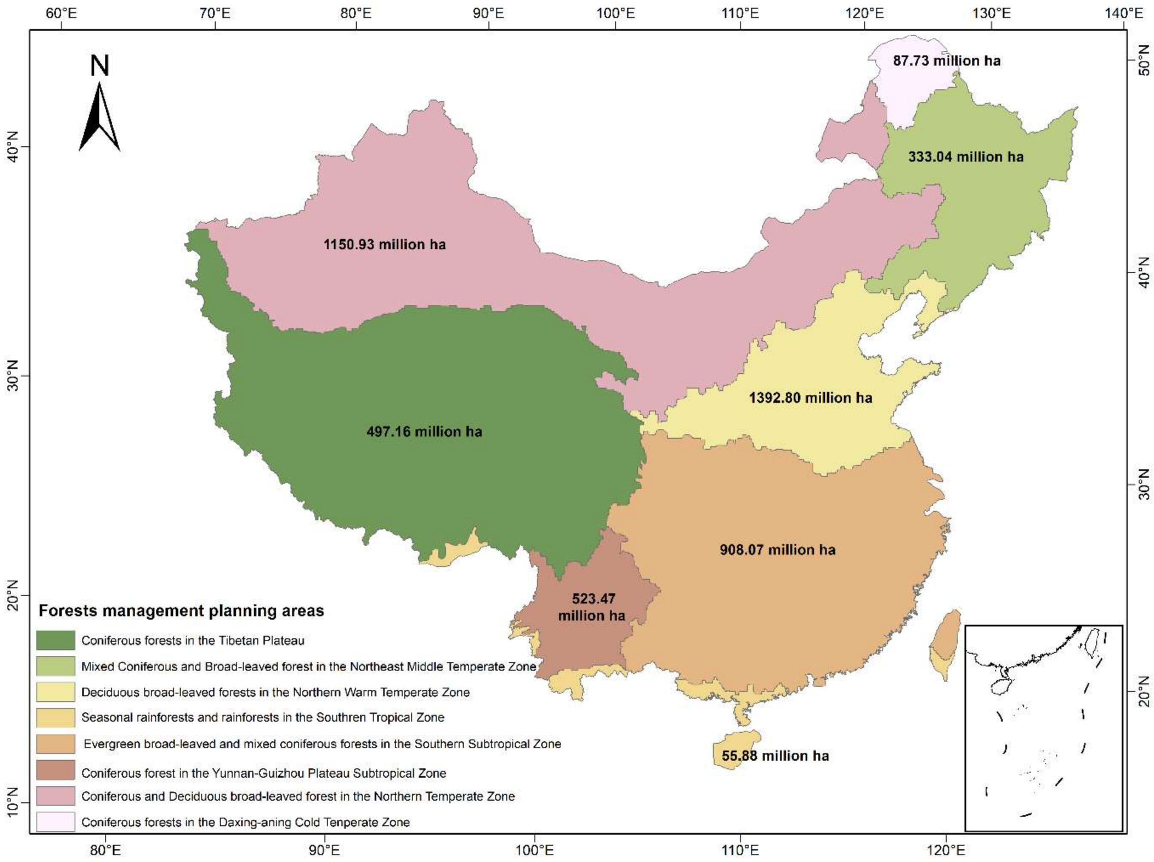

Fig. S17. The forest management zones designed by the state forestry administration. The planned forestation areas were showed in each zones.

Spatial distribution of tree replacement practice

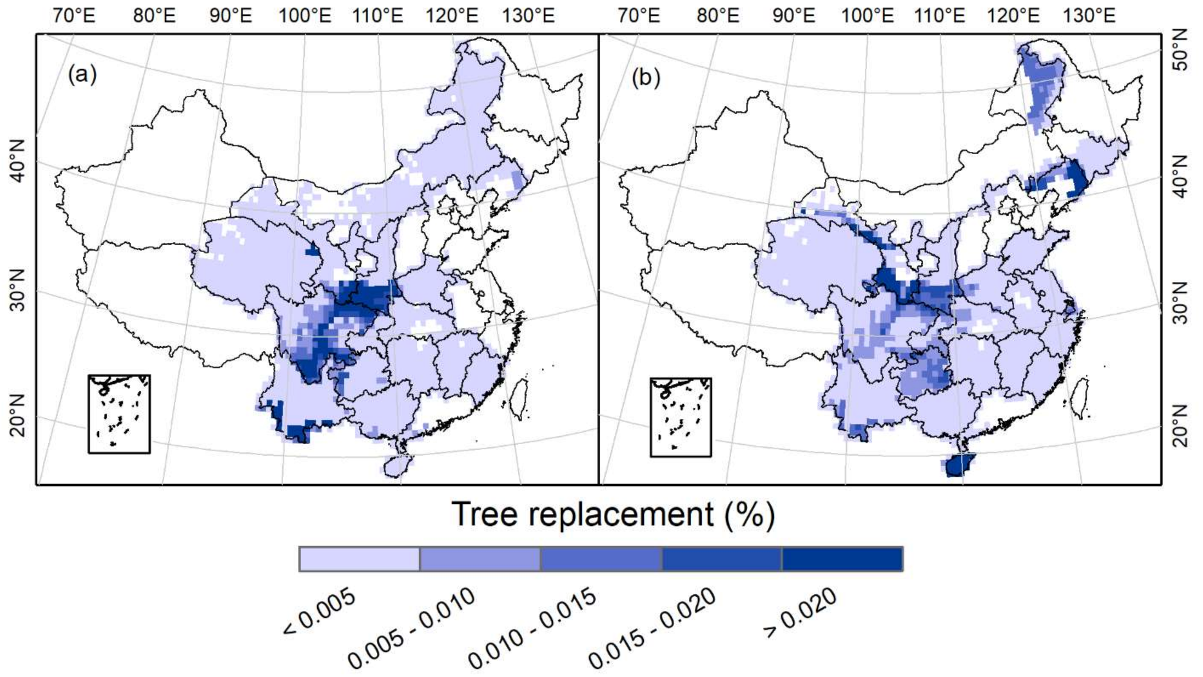

Fig. S18. Spatial distribution of tree replacement practice. Panel a: natural forest; Panel b: planted forest; the value indicates the percent of trees to be replaced in each grid cell.

Table S1. Projected carbon stock and sequestration rate reported in China's forests

Approach	Stock (Pg C)		Year	Source	This study
	Initial*	Increase**			
Statistical model	16.47	4.16	2040s	(2)	4.54
	7.63-10.32	2.34-5.52	2050	(3–6)	5.38
	8.79	12.03	2063	(7)	7.76
Process-based model	7.07	13.86	2100	(8)	13.6±1.5
	20.79	4.21	2100	(9)	
**Approach**	**Sink*** (Pg C yr^{-1})**		**Period**	**Source**	**This study**
Statistical model	0.105-0.236		2020-2040	(2, 3)	0.173±0.017
	0.198-0.300		2020-2050	(10, 11)	0.178±0.016
	0.124-0.253		2020-2060	(12–14)	0.179±0.014
	0.20		2020-2100	(15)	0.172±0.04
Process-based model	0.106-0.209		2020-2100	(8, 9)	0.172±0.04

*Initial carbon stock in 2020; **Carbon stock increment from 2020 to the last year/decade reported. Carbon stock Increment in Zhang et al (2022) is abnormally high due to unrealistic increase of forest area; ***Carbon sinks of the periods between 2020 and the last reported year were listed.

Table S2. Biomass expansion factor (BEF) and its parameters for China's major tree species. ($BEF = a + b/x$, where *a* and *b* are constant, *x* is mean stock volume per hectare).

Forest type	Parameters		Reference
	a	b	
Abies spp.	0.5519	48.861	(16)
Picea spp.	0.5519	48.861	(16)
Tsuga spp.	0.4158	41.3318	(17)
Keteleeria	0.4158	41.3318	(17)
Larix spp.	0.6096	33.806	(16)
Pinus koraiensis	0.5723	16.4890	(16)
Pinus sylvestris	1.1120	2.6951	(16)
Pinus densiflora	1.0945	2.004	(16)
Pinus thunbergii	0.8200	16.414	(18)
Pinus tabulae	0.869	9.1212	(16)
Pinus armandii	0.5856	18.7440	(16)
Pinus massoniana	0.5034	20.5470	(16)
Pinus yunnanensis	0.5034	20.5470	(16)
Pinus kesiya	0.5101	1.0451	(16)
Pinus densata	0.8100	11.892	(18)
Foreign pine (other pines introduced from other countries)	0.5292	25.087	(16)
Pinus elliottii	0.6800	19.7590	(18)
Pinus taiwanensis	0.5168	33.2378	(16)
Other pines and conifer forests	0.5292	25.087	(16)
Cunninghamia lanceolata	0.4652	19.141	(16)
Cryptomeria	0.3491	39.816	(16)
Metasequoia glyptostroboides	0.4158	41.3318	(19)
Cupressus funebris Endl	0.8893	7.3965	(16)
Quercus spp.	1.1453	8.5473	(17)
Betula	1.0687	10.237	(16)

Betula platyphylla	1.3300	-2.881	(18)
Betula costata	0.9300	16.459	(18)
Fraxinus mandshurica	0.7564	8.3103	(20)
Juglans mandshurica	0.7564	8.3103	(20)
Cinnamomum longipaniculatum	1.0357	8.0591	(17)
Phoebe zhennan	1.0357	8.0591	(17)
Ulmus	0.7564	8.3103	(20)
Robinia pseudoacacia	0.7564	8.3103	(21)
Schima	0.9200	19.808	(18)
Other hardwood broadleaf species	0.9600	29.083	(18)
Tilia tuan	0.6800	54.484	(18)
Sassafras	0.9788	5.3764	(16)
Populus spp.	0.4969	26.973	(16)
Salix babylonica	0.5100	44.003	(18)
Eucalyptus spp.	0.8873	4.5539	(16)
Acacia yunnanensis	0.8100	10.371	(18)
Casuarina	0.7441	3.2377	(16)
Other softwood broadleaf forests	0.6200	33.931	(18)
Mixed Needle-leaf forests	0.5292	25.087	(16)
Mixed Broadleaf forests	0.6255	8.3103	(17)
Mixed Needle-leaf and broadleaf forests	0.8136	18.466	(17)

Table S3. Biomass-carbon conversion coefficients for different species

Forest type	Coefficient	Reference	Forest type	Coefficient	Reference
Abies spp.	0.4990	(16)	*Quercus spp.*	0.5004	(17)
Picea spp.	0.5208	(16)	*Butula*	0.4914	(16)
Tsuga spp.	0.5022	(17)	*Betula platyphylla*	0.4914	(18)
Keteleeria	0.5207	(17)	*Betula costata*	0.4914	(18)
Larix spp.	0.5211	(16)	*Fraxinus mandshurica*	0.4827	(20)
Pinus koraiensis	0.5113	(16)	*Juglans mandshurica*	0.4827	(20)
Pinus sylvestris	0.5223	(16)	*Cinnamomum longipaniculatum*	0.4916	(17)
Pinus densiflora	0.5141	(17)	*Phoebe zhennan*	0.503	(17)
Pinus thunbergii	0.5146	(18)	*Ulmus*	0.5000	(20)
Pinus tabulae	0.5207	(16)	*Robinia pseudoacacia*	0.5000	(21)
Pinus armandii	0.5225	(16)	*Schima*	0.5000	(18)
Pinus massoniana	0.4596	(16)	Other hardwood and broadleaf forests	0.4834	(18)
Pinus yunnanensis	0.5113	(16)	*Tilia tuan*	0.4392	(18)
Pinus kesiya	0.5224	(17)	*Sassafras*	0.4848	(16)
Pinus densata	0.5009	(18)	*Populus spp.*	0.4956	(16)
Pinus elliottii	0.5000	(18)	*Eucalyptus spp.*	0.5253	(16)
Pinus taiwanensis	0.5000	(16)	*Acacia yunnanensis*	0.500	(18)
Other foreign *Pinus*	0.5000	(16)	*Salix babylonica*	0.5000	(18)
Other pines and conifer forests	0.5000	(16)	*Casusrina*	0.4980	(17)
Cunninghamia lanceolata	0.5201	(16)	Other softwood and broadleaf forests	0.4956	(18)
Cryptomeria	0.5235	(16)	Needle-leaf mixed forests	0.5111	(16)
Metasequoia glyptostroboides	0.5013	(19)	Broadleaf mixed forests	0.4900	(17)
Cupressus funebris Endl	0.5034	(16)	Needle-leaf and broadleaf mixed forests	0.5009	(17)

Table S4. The tree species and the number of forest site-records (plots) used in this study

	Natural forest		Planted forest	
	**species**	**Sample size**	**species**	**Sample size**
Needle-leaf forest	*Abies*	3243	*Abies*	38
	Cryptomeria fortunei	22	*Cryptomeria fortunei*	403
	Cunninghamia lanceolata	3269	*Cunninghamia lanceolata*	11223
	Cupressus	4153	*Cupressus*	1853
	Keteleeria	218	Foreign pines (other pines introduced from other countries)	276
	Larix	6080	*Larix*	3586
	Other pines	107	*Metasequoia glyptostroboides*	207
	Picea	9255	Other pines	172
	Pinus armandii	491	*Picea*	482
	Pinus densata	3589	*Pinus armandii*	510
	Pinus densiflora	190	*Pinus densata*	28
	Pinus kesiya var. *langbiamensis*	399	*Pinus densiflora*	143
	Pinus koraiensis	79	*Pinus elliottii*	842
	Pinus massoniana	10942	*Pinus kesiya* var. *langbiamensis*	80
	Pinus sylvestris	215	*Pinus koraiensis*	256
	Pinus tabulaeformis	1788	*Pinus massoniana*	4815
	Pinus taiwanensis	116	*Pinus sylvestris*	412
	Pinus yunnanensis	4560	*Pinus tabulaeformis*	2928
	Tsuga	122	*Pinus taeda*	69
			Pinus taiwanensis	37
			Pinus thunbergii	366
			Pinus yunnanensis	388
			Taxodium ascendens	26
Broadleaf forest	*Betula*	4149	*Acacia*	221
	Betula costata	224	*Betula*	64
	Betula platyphylla	5053	*Camphora officinarum*	358
	Camphora officinarum	61	*Casuarina equisetifolia*	137
	F.J.P *	180	*Eucalyptus*	2972
	Fraxinus mandshurica	64	*Liquidamber*	68

	Juglans mandshurica	332	*Melia azedarach*	27
	Liquidamber	202	Other hardwood broadleaf species	2111
	Other hardwood broadleaf species	7151	Other softwood broadleaf species	1296
	Other softwood broadleaf species	4537	*Paulawnia fortunei*	335
	Paulawnia fortunei	24	*Populus*	13023
	Phoebe	86	*Quercus*	771
	Populus	2157	*Robinia pseudoacacia*	1453
	Quercus	19315	*Salix*	453
	Robinia pseudoacacia	33	*Sassafras*	23
	Salix	160	*Schima superba*	94
	Schima superba	287	*Ulmus pumila*	345
	Tilia	669		
	Ulmus pumila	605		
Mixed forest	Mixed broadleaf forest (MBF)	41675	Mixed broadleaf forest (MBF)	1978
	Mixed needle-leaf and broadleaf forest (MNBF)	8229	Mixed needle-leaf and broadleaf forest (MNBF)	6777
	Mixed needle-leaf forest (MNF)	3198	Mixed needle-leaf forest (MNF)	1889

*F.J.P denotes the abbreviation of *Fraxinus mandshurica*, *Juglans mandshurica* and *Phellodendrom amurense*. These three tree species were reported together.

Table S5. Experiments designed for model simulations

Group	Abbreviation	Drivers/settings				
		Tree survival in new PF	Wood harvest	Rotation age*	Tree replacement	Climate and CO_2
1	S1	47%	Yes	Regular	No	SSP126
	S2	47%	Yes	Regular	No	SSP245
	S3	47%	Yes	Regular	No	SSP370
	S4	47%	Yes	Regular	No	SSP585
	S5	85%	Yes	Regular	No	SSP126
	S6	85%	Yes	Regular	No	SSP245
	S7	85%	Yes	Regular	No	SSP370
	S8	85%	Yes	Regular	No	SSP585
2	S9	47%	Yes	Regular+5N	No	SSP126
	S10	47%	Yes	Regular+5N	No	SSP245
	S11	47%	Yes	Regular+5N	No	SSP370
	S12	47%	Yes	Regular+5N	No	SSP585
	S13	47%	Yes	Regular	Yes	SSP126
	S14	47%	Yes	Regular	Yes	SSP245
	S15	47%	Yes	Regular	Yes	SSP370
	S16	47%	Yes	Regular	Yes	SSP585
	S17	47%	Yes	Regular+5N	Yes	SSP126
	S18	47%	Yes	Regular+5N	Yes	SSP245
	S19	47%	Yes	Regular+5N	Yes	SSP370
	S20	47%	Yes	Regular+5N	Yes	SSP585
	S21	85%	Yes	Regular+5N	No	SSP126
	S22	85%	Yes	Regular+5N	No	SSP245
	S23	85%	Yes	Regular+5N	No	SSP370
	S24	85%	Yes	Regular+5N	No	SSP585

	S25	85%	Yes	Regular	Yes	SSP126
	S26	85%	Yes	Regular	Yes	SSP245
	S27	85%	Yes	Regular	Yes	SSP370
	S28	85%	Yes	Regular	Yes	SSP585
	S29	85%	Yes	Regular+5N	Yes	SSP126
	S30	85%	Yes	Regular+5N	Yes	SSP245
	S31	85%	Yes	Regular+5N	Yes	SSP370
	S32	85%	Yes	Regular+5N	Yes	SSP585
3	S33	47%	No	-	No	Fixed at 2020
	S34	85%	No	-	No	Fixed at 2020
4**	S35	47%	Yes	Regular	No	SSP126
	S36	47%	Yes	Regular	No	SSP245
	S37	47%	Yes	Regular	No	SSP370
	S38	47%	Yes	Regular	No	SSP585
	S39	85%	Yes	Regular	No	SSP126
	S40	85%	Yes	Regular	No	SSP245
	S41	85%	Yes	Regular	No	SSP370
	S42	85%	Yes	Regular	No	SSP585

*'Regular' in rotation age indicate the wood harvest rotation age is species-based obtained from the State Forestry Administration of China (i.e., Regulations for age-class and age-group division of main tree-species), while 'Regular+5N' indicate the harvest age is postponed by 5*N years after key year (i.e. year that timber forest shows the largest carbon source), and N is 1, 2, or 3; in total of 56 experiments (i.e. 7 management scenarios × 2 tree survival rate scenarios × 4 SSPs) were conducted in group 2; nitrogen deposition was fixed since 2015, because a former study revealed that overall nitrogen deposition has been stabilized due to improved agricultural and environmental policies (22). ** Group 4 was designed similar as group 1 but the land use and cover change was fixed at 2020.

Table S6. Harvest age of different tree species/species groups

Tree species/species group	Natural forest		Planted forest	
	Age1*	Age2**	Age1	Age2
Pinus massoniana	37	51	26	36
Cunninghamia lanceolata	19	26	19	26
Eucalyptus	12	16	12	16
Populus	30	41	14	18.5
Larix	67	91	30	41
Pinus tabulaeformis	37	51	26	36
Cupressus	74	101	45	61
Pinus elliottii	19	26	19	26
Robinia pseudoacacia	6	18.5	6	18.5
Other *Pinus*	37	51	26	36
Mixed broadleaf forest	36	48.5	36	48.5
Other softwood broadleaf species	34	46	20	27.25
Other hardwood broadleaf species	37	51	26	36
Mixed needle-leaf and broadleaf forest	45	61	25	34.75
Mixed needle-leaf forest	29	38.5	23	31
Quercus	44	60	36	50
Abies, Picea	59	81	37	51
Pinus armandii, Pinus yunnanensis	67	91	30	41
Economic forests	26	36	26	36
Bamboo	7	10	7	10
Betula	20	55	40	36
Other temperate deciduous forest species	30	41	30	41
Other fir species	59	80	59	80

*Age1: the age at the peak of the instantaneous growth rate; **Age2: the harvest age, which is also the peak of the accumulated growth rate (i.e. the average of instantaneous growth rate).

Table S7. Parameter *a* of different tree species/species groups in the logistic equation of age factor

Tree species/species group	Natural forest	Planted forest
Pinus massoniana	0.15	0.05
Cunninghamia lanceolata	0.05	0.25
Eucalyptus	0.05	0.05
Populus	0.05	0.05
Larix	0.05	0.20
Pinus tabulaeformis	0.05	0.05
Cupressus	0.05	0.25
Pinus elliottii	0.05	0.05
Robinia pseudoacacia	0.05	0.05
Other *Pinus*	0.05	0.05
Mixed broadleaf forest	0.35	0.35
Other softwood broadleaf species	0.35	0.35
Other hardwood broadleaf species	0.05	0.05
Mixed needle-leaf and broadleaf forest	0.05	0.25
Mixed needle-leaf forest	0.25	0.15
Quercus	0.05	0.05
Abies, Picea	0.20	0.05
Pinus armandii, Pinus yunnanensis	0.05	0.15
Economic forests	0.05	0.05
Bamboo	0.05	0.05
Betula	0.05	0.05
Other temperate deciduous forest species	0.05	0.05
Other fir species	0.05	0.05

Table S8 Forest age increment in non-timber forests with mixed tree species

Tree species/species group*	Harvest age			
	Natural forest		Planted forest	
	Age accrual per year	Std	Age accrual per year	Std
Mixed broadleaf forest	0.848	0.358	0.788	0.284
Other softwood broadleaf species	0.924	0.154	0.872	0.230
Other hardwood broadleaf species	0.828	0.292	0.890	0.488
Mixed needle-leaf and broadleaf forest	0.790	0.302	0.794	0.250
Mixed needle-leaf forest	0.832	0.210	0.820	0.258

Table S9. Tree species replacement plan

Management zone	Inappropriate species	Area (km^2)	Replacement species
Needleleaf forests of cold temperate zone	*Pinus tabulae*	947.2	*Larix*
	Cupressus funebris Endl	52.5	
	Robinia pseudoacacia	49.2	
Needleleaf&broadleaf mixed forests of mid-temperate zone	*Pinus tabulae*	3039.3	*Larix*
	Cupressus funebris Endl	94.1	
	Pinus elliottii	15.9	*Pinus koraiensis*
	Robinia pseudoacacia	751.4	*Larix*
Deciduous broadleaf forests of warm-temperate zone	*Pinus massoniana*	372.7	*Pinus koraiensis*
	Cunninghamia lanceolata Hook	104.4	
	Pinus elliottii	35.8	*Larix*
Broadleaf evergreen and needleleaf-broadleaf mixed forests of subtropical zone	*Larix*	1479.8	BMF species*
	Pinus tabulae	4089.9	
	Betula	4206.1	
	Other firs	4474.0	
Tropical and seasonal tropical zone	*Populus*	24.4	BMF species
	Larix	5.5	
	Cupressus funebris Endl	63.7	
	Robinia pseudoacacia	0.2	
	Quercus	6102.5	
	Picea	258.1	
	Pinus armandii Franch	2795.2	
	Betula	91.2	
	Other firs	214.6	
Needleleaf forest of subtropical zone	*Pinus massoniana*	1338.9	*Pinus armandii* Franch
	Pinus tabulae	189.2	
	Robinia pseudoacacia	94.7	BMF species
	Betula	1633.4	
Needleleaf and deciduous broadleaf forest of temperate zone	*Pinus massoniana*	30.8	*Larix*
	Cunninghamia lanceolata Hook	7.4	*Picea*
	Cupressus funebris Endl	1387.5	*Larix*
	Robinia pseudoacacia	24.6	BMF species
	Betula	5.1	
Needleleaf forest of Tibetan Plateau	*Pinus massoniana*	1454.5	*Picea*
	Cunninghamia lanceolata Hook	590.8	*Picea*
	Pinus tabulae	1415.2	*Pinus armandii* Franch
	Pinus elliottii	29.5	*Pinus densata*
	Robinia pseudoacacia	3902.0	BMF species

*BMF species indicates Broadleaf mixed forest species, which are indigenous species of southern China, these species were grouped into one type in model simulations.

Supplementary References

1. Minstry of Agriculture and Forestry of China, *Timber Volume Table of China* (Standards Press of China, 1978).
2. Y. Yao, S. Piao, T. Wang, Future biomass carbon sequestration capacity of Chinese forests. *Sci. Bull.* **63**, 1108–1117 (2018).
3. Q. Li, J.-H. Zhu, Y. Feng, W.-F. Xiao, Carbon storage and carbon sequestration potential of the forest in China. *Clim. Chang. Res.* **14**, 287–294 (2018).
4. B. Xu, Z. Di Guo, S. L. Piao, J. Y. Fang, Biomass carbon stocks in China's forests between 2000 and 2050: A prediction based on forest biomass-age relationships. *Sci. China Life Sci.* **53**, 776–783 (2010).
5. H. Hu, S. Wang, Z. Guo, B. Xu, J. Fang, The stage-classified matrix models project a significant increase in biomass carbon stocks in China's forests between 2005 and 2050. *Sci. Rep.* **5**, 11203 (2015).
6. Z. Qiu, Z. Feng, Y. Song, M. Li, P. Zhang, Carbon sequestration potential of forest vegetation in China from 2003 to 2050: Predicting forest vegetation growth based on climate and the environment. *J. Clean. Prod.* **252**, 119715 (2020).
7. Y. Zhang, X. Li, Y. Wen, Forest carbon sequestration potential in China under the background of carbon emission peak and carbon neutralization. *J. Beijing For. Univ.* **44**, 38–47 (2022).
8. L. Zhou, *et al.*, Carbon dynamics in woody biomass of forest ecosystem in China with forest management practices under future climate change and rising CO_2 concentration. *Chinese Geogr. Sci.* **23**, 519–536 (2013).
9. W. M. Ju, J. M. Chen, D. Harvey, S. Wang, Future carbon balance of China's forests under climate change and increasing CO_2. *J. Environ. Manage.* **85**, 538–562 (2007).
10. N. He, *et al.*, Vegetation carbon sequestration in Chinese forests from 2010 to 2050. *Glob. Chang. Biol.* **23**, 1575–1584 (2017).
11. C. Zhang, *et al.*, Sustained biomass carbon sequestration by China's forests from 2010 to 2050. *Forests* **9**, 689 (2018).
12. S. Piao, C. Yue, J. Ding, Z. Guo, Perspectives on the role of terrestrial ecosystems in the "carbon neutrality" strategy. *Sci. China Earth Sci.* **65**, 1178–1186 (2022).

13. W. Cai, *et al.*, Carbon sequestration of Chinese forests from 2010 to 2060: spatiotemporal dynamics and its regulatory strategies. *Sci. Bull.* **67**, 836–843 (2022).
14. Y. Huang, *et al.*, The role of China's terrestrial carbon sequestration 2010-2060 in offsetting energy-related CO_2 emissions. *Natl. Sci. Rev.* **9**, nwac057 (2022).
15. H. Xu, C. Yue, Y. Zhang, D. Liu, S. Piao, Forestation at the right time with the right species can generate persistent carbon benefits in China. *Proc. Natl. Acad. Sci. U. S. A.* **120**, e2304988120 (2023).
16. S. L. Piao, *et al.*, The carbon budget of terrestrial ecosystems in East Asia over the last two decades. *Biogeosciences* **9**, 3571–3586 (2012).
17. H. Li, Y. Lei, *Estimation and Evaluation of Forest Biomass Carbon Storage in China* (China Forestry Publishing House, 2010).
18. J. Lu, "Estimation of carbon storage of arbor forest based on the forest resource inventory data," Beijing Forestry University. (2020).
19. G. Liu, B. Fu, J. Fang, Carbon dynamics of Chinese forests and its contribution to global carbon balance. *Acta Ecol. Sin.* **20**, 733–740 (2000).
20. Q. Yang, Estimating forest carbon storage in Xiuyan County by forest stock volume biomass model. *Green Technol.* **23**, 20–22 (2021).
21. J. Fang, G. Liu, S. Xu, Biomass and net production of forest vegetation in China. *Acta Ecol. Sin.* **16**, 497–508 (1996).
22. G. Yu, *et al.*, Stabilization of atmospheric nitrogen deposition in China over the past decade. *Nat. Geosci.* **12**, 424–429 (2019).
