# Supplementary material for: Maximizing carbon sequestration potential in Chinese forests through optimal management
Source: Nat Commun. 2024 Apr 11;15:3154. doi: 10.1038/s41467-024-47143-5 (PMC11009231; doi:10.1038/s41467-024-47143-5)
Supplement: Supplementary file 3 — Description of Additional Supplementary Files [file 41467_2024_47143_MOESM3_ESM.pdf]

Description of Additional Supplementary Files

File Name: Supplementary Data 1
Description: equations of the statistical models
